# Hippocampal Administration of Levothyroxine Impairs Contextual Fear Memory Consolidation in Rats

**DOI:** 10.3389/fncel.2017.00223

**Published:** 2017-07-31

**Authors:** Dafu Yu, Heng Zhou, Lin Zou, Yong Jiang, Xiaoqun Wu, Lizhu Jiang, Qixin Zhou, Yuexiong Yang, Lin Xu, Rongrong Mao

**Affiliations:** ^1^Department of Nuclear Medicine, First People’s Hospital of Yunnan Province Kunming, China; ^2^Laboratory of Learning and Memory, Kunming Institute of Zoology, Chinese Academy of Sciences Kunming, China; ^3^School of Life Sciences, University of Science and Technology of China Hefei, China; ^4^Department of Laboratory Medicine, The First Affiliated Hospital of Chongqing Medical University Chongqing, China; ^5^Respiratory Department, First People’s Hospital of Yunnan Province Kunming, China; ^6^Department of Neuropsychopathy, Clinical Medical School, Dali University Dali, China

**Keywords:** levothyroxine, hippocampus, fear memory, consolidation, acquisition, retrieval

## Abstract

Thyroid hormone (TH) receptors are highly distributed in the hippocampus, which plays a vital role in memory processes. However, how THs are involved in the different stages of memory process is little known. Herein, we used hippocampus dependent contextual fear conditioning to address the effects of hippocampal THs on the different stages of fear memory. First, we found that a single systemic levothyroxine (LT_4_) administration increased the level of free triiodothyronine (FT_3_) and free tetraiodothyroxine (FT_4_) not only in serum but also in hippocampus. In addition, a single systemic LT_4_ administration immediately after fear conditioning significantly impaired fear memory. These results indicated the important role of hippocampal THs in fear memory process. To further confirm the effects of hippocampal THs on the different stages of fear memory, LT_4_ (0.4 μg/μl, 1 μl/side) was injected bilaterally into hippocampus. Rats given LT_4_ into hippocampus before training or tests had no effect on the acquisition or retrieval of fear memory, however rats given LT_4_ into hippocampus either immediately or 2 h after training showed being significantly impaired fear memory, which demonstrated LT_4_ administration into hippocampus impairs the consolidation but has no effect on the acquisition and retrieval of fear memory. Furthermore, hippocampal injection of LT_4_ did not affect rats’ locomotor activity, thigmotaxis and THs level in prefrontal cortex (PFC) and serum. These findings may have important implications for understanding mechanisms underlying contribution of THs to memory disorders.

## Introduction

Thyroid hormones (THs), triiodothyronine (T_3_) and thyroxine (T_4_), regulate some target genes transcriptions through TH receptors α (TRα) or β (TRβ) in cell nucleus (Cheng et al., 2010) or nongenomic actions in cytoplasm (Davis et al., 2016). Type 2 deiodinase enzyme is responsible for the conversion of T_4_ to nuclear active T_3_ (Baumgartner et al., 1998). THs play critical roles in the regulation of neurogenesis, neuronal proliferation and migration, dendritic branching and synaptogenesis, glial differentiation and migration, axonal outgrowth and myelination, brain maturation and cognitive functions (Williams, 2008; Raymaekers and Darras, 2017) and so on. Homeostatic equilibria of THs depend on dynamic inter-relationships among THs, pituitary thyrotropin and hypothalamic thyrotropin-releasing hormone (Hoermann et al., 2015, 2016). THs dysfunctions are accompanied by some neurological (Raymaekers and Darras, 2017) and psychiatric disorders, as well as cognitive impairments such as abnormal working memory in some patients with Graves’ disease (Jabłkowska et al., 2008) or subclinical hypothyroidism (Beydoun et al., 2015), the autobiographical memory deficits in patients with early TH deficiency (Willoughby et al., 2014). Animal study proves that both systemic levothyroxine (LT_4_) injection and hypothyroidism impaired spatial memory of rats in Y-maze (Taşkin et al., 2011; Artis et al., 2012). Neonatal rats treated with T_3_ showed impaired spatial memory and synaptic long-term potentiation (LTP) induction in hippocampus (Pavlides et al., 1991). However, intracerebroventricular administration of T_3_ before retrieval can improve passive avoidance memory in rat model of ischemic brain stroke (Mokhtari et al., 2017). Daily intraperitoneally injected with LT_4_ for 3 months restored impaired spatial memory in aged mice (Fu et al., 2014). Supplementation with T_3_ or T_4_ 2 weeks before training blocked the enhancement of fear memory in a hypothyroid mouse model (Buras et al., 2014). However, the role of THs in the different stages of memory, which will strengthen understanding the mechanisms underlying contribution of THs to memory disorders, is still poorly understood. In this study, we used systemic and hippocampal administration of LT_4_, which is widely used to treat hypothyroidism in clinic and can be converted into T_3_ in euthyroid rats, to explore the role of THs in the different stages of memory.

The hippocampus, a TH receptor-rich region (Singh et al., 2016), plays vital roles in memory processes. Several findings indicate that THs regulate hippocampus-dependent memory processes. In clinic, patients with hyperthyroid disorders show being decreased gray matter volumes in hippocampus (Zhang et al., 2014). Meanwhile, some patients with congenital hypothyroidism show abnormal hippocampal function and verbal memory (Wheeler et al., 2015). THs replacement treatment improves hippocampus-dependent learning and memory in hypothyroid animals (Ge et al., 2015) and humans (Miller et al., 2006). T_3_ administration in hippocampus enhances long-term memory for trace cued and delays contextual fear conditioning in rats (Sui et al., 2006).

Here, we aim to investigate the effects of LT_4_ administrated into hippocampus on the different stages of fear memory process.

## Materials and Methods

### Animals

Male Sprague-Dawley rats (Animal House Center, Kunming Medical University, China), weighing 250–300 g, 11–12 weeks old, were used. Rats were individually housed in home cages with *ad libitum* access to water and food (complying with the Chinese rat food standard GB 14924.3-2001) and subjected to a 12-h light/12-h dark cycle (lights on at 7 am) in a temperature-regulated room. Rats were allowed 1 week to acclimate our research facility before manipulations. All experiments were performed between 09:00 and 12:00. This study was carried out in accordance with the recommendations of the National Institutes of Health on experimental animal care and use. All experimental protocols were approved by the Animal Ethics Committee of the Kunming Institute of Zoology, Chinese Academy of Sciences.

### Cannula Implantation and Drug Infusion

The protocol of cannula implantation was similar to our previous study (Zhou et al., 2016). Surgery was performed under the anesthesia of pentobarbital sodium salt (Sigma, USA, dissolved in saline, 60 mg/kg body weight, I.P.). Rats were ventilated with 95% O_2_/5% CO_2_ through a mask and mounted in a stereotaxic apparatus (RWD Life Science Co., Shenzhen, China). Small holes were bilaterally drilled in the skull for the placement of two stainless-steel guide cannulae (7 mm length, 26 gauges) in the dorsal hippocampus (DH). The cannula tips were targeted to 1 mm above the DH with the coordinate of anteroposterior = −3.8 mm from the bregma, mediolateral = ±2.8 mm, dorsoventral = −3.0 mm. Cannula were affixed to the skull using dental cement. A stylet was introduced into the guide cannula to prevent possible obstruction. The rats were allowed to recover for 7 days following surgery and handled for 3 days before behavioral experiments. To test the role of hippocampal THs in our behavioral paradigm, LT_4_ solution (Sigma, 0.4 μg/μl, saline as vehicle, 1 μl/side) was bilaterally injected into the hippocampus (I.H.) using a microsyringe pump at the speed of 0.2 μl/min with an injection pipe via cannula. After all behavioral tests, an injection pipe used in the experiments was inserted into the cannula for infusing 1 μl trypan blue as a marker to test the accuracy of cannula placement in each rat.

### FT_4_, FT_3_ Levels in Serum or Brain Tissues

The rats were anesthetized using ether at all scheduled time points after intraperitoneal drug administration (I.P.), and their heart blood samples were obtained to test serum levels of free tetraiodothyroxine (FT_4_) and free triiodothyronine (FT_3_). For collecting brain tissues, rats were perfused from left ventricle with saline 500 ml for 30 min after anesthesia of pentobarbital sodium salt (Sigma, 60 mg/kg body weight). Immediately at the end of saline perfusion, dissection of their hippocampus and prefrontal cortices (PFC) were followed (Chiu et al., 2007). Sample of bilateral hippocampus or PFC was individually homogenized in artificial cerebral spinal fluid (ACSF; Mahmoud and Amer, 2014) by the ratio of 1 μg/4 μl (brain tissues/ACSF) in an ice surrounded homogenizer and centrifuged at 14,000 rpm for 15 min at 4°C. The resulting supernatant was collected and stored at −80°C until the determination of FT_4_ and FT_3_.

FT_4_ and FT_3_ were tested using time-resolved fluoroimmunoassays (TRFIA). Diagnostic kits for FT_4_ and FT_3_ (SYM Bio Lifescience Co., LTD, Suzhou, China) were used in conjunction with a multilabel counter (Wallac Vicotor 2 TM 1420 multilabel counter, Turku, Finland) to detect THs levels according to the manufacturer’s instructions. The detection ranges for FT_4_ and FT_3_ were 0–74 pmol/L and 0–54 pmol/L, respectively. Whenever the concentration of a particular hormone exceeded the corresponding upper limit, the serum was further diluted with saline to achieve a measurable concentration. All tests were done in triplicate.

### The Open Field Test

Locomotor activity and thigmotaxis of rats were tested in an open field at 24 H following LT_4_ or saline administration (I.P. or I.H., saline as vehicle). The rats were individually placed in the center of an open field (43 cm × 43 cm × 30 cm with virtual central zone in it, ENV-017M-027, Med Associates, Inc., St. Albans, VT, USA) and allowed to explore freely for 20 min. Rat movement was tracked using three 16-beam infrared arrays around the rat arena. The open field was cleaned with 5% ethanol after each test. The running distance reflected the locomotor activity and was automatically analyzed using the Med Associates software after test. Thigmotaxis was presented as the percentage (%) of the total distance moved (TDM) in the central zone of the test apparatus. Specifically, thigmotaxis was calculated as the ratio between TDM in the central zone and TDM over the whole test arena (including central and outer zones).

### The Treadmill Test

The protocol was similar to our previous study (Yu et al., 2015). The rats were placed onto a treadmill apparatus (Panlab Technology, Spain) following the same time window of LT_4_ or saline administration as that in the open field tests. The running intensity was set at 25 m/min with no incline. During a 5 min test, the running distance, which reflected fatigue resistance ability, was automatically recorded.

### Histology

At completion of behavioral testing, the rats were administered (I.P.) an overdose of pentobarbital sodium (Sigma), infused 1 μl trypan blue into bilateral hippocampus through the guide cannula with the same injection needle tip depth as LT_4_ or saline infusion, and perfused transcardially with saline followed by 4% formaldehyde solution (in 0.1 M phosphate buffer). After extraction from the skull, the brains were removed and cryoprotected in 20% glycerol/10% formaldehyde solution for 3 days. Coronal sections (50 μm thickness) were cut through hippocampus with a freezing microtome (VT1000 S, Leica Biosystems, Germany), and wet-mounted on glass microscope slides with 70% ethanol. The nature and extent of the hippocampal lesion was verified by visual inspection of the stained brain sections. All rats had complete, bilateral lesions of the DH with sparing of axonal fibers of passage. All hippocampal lesions in this experiment were virtually identical.

### Contextual Fear Conditioning

The protocol was similar to that in our previous studies (Zhou et al., 2016). During the training process, rats were placed in a chamber (32 × 25 × 25 cm; Med Associates Inc., St. Albans, VT, USA), allowed to freely explore the box for 2 min, and then received 5-trial foot shocks (0.8 mA, 2 s) with 2-min intervals. The freezing time, which reflected the expression of the fear response to the context or aversive stimulus, was automatically recorded by software (Video Freeze V2.5.5.0, Med Associates, Inc., St. Albans, VT, USA) before the first conditioning trial (Pre), and the freezing times were again measured during the 2-min interval after each conditioning trial to reflect the level of acquisition. To test contextual memory, the rats were placed back in the box for 5 min without foot shocks on day 1 (test 1), day 7 (test 2), and day 14 (test 3) post training. The freezing time was recorded to represent the level of memory. All experimenters were blinded to the group assignment of the animals.

To test the systemic effects of THs on fear memory, rats were injected (I.P.) with LT_4_ (Sigma, 15 μg/ml × 1 ml/kg body weight) immediately after fear conditioning. To test the contributions of THs in hippocampus to fear memory, rats were directly injected with LT_4_ into DH 1 day before, immediately, 2 h or 23.5 h after fear conditioning. All control rats were injected saline (1 ml/kg body weight during systemic LT_4_ administration or 1 μl/side hippocampus during hippocampal LT_4_ administration) with the same injection sites as experimental rats.

### Statistical Analysis

The percent time freezing (total freezing time/120 s × 100%) every 120 s in six consecutive observations during fear conditioning or (total freezing time/300 s × 100%) during the fear memory test for 300 s for each rat was calculated. All data were represented as mean ± SEM. Independent *Student*’s *t*-test, repeated measures ANOVA, and one-way ANOVA followed by *post hoc* Bonferroni’s test were used for statistical analyses according to experimental designs (SPSS 16.0 version). *P* < 0.05 was considered to be statistically significant.

## Results

### The Effects of a Single Systemic Injection of LT_4_ on THs in Serum and Hippocampus, and Contextual Fear Memory

**Figure 1:**
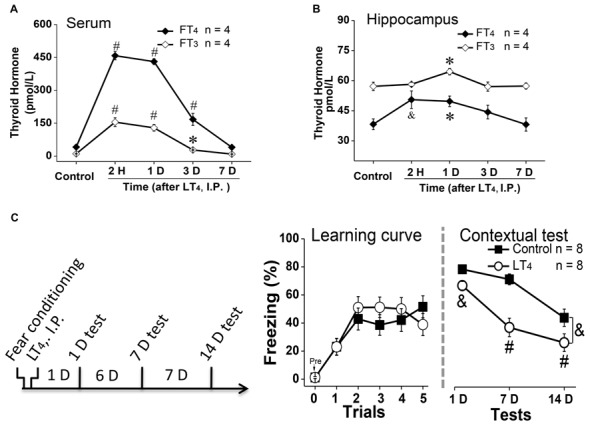
Effects of systemic injection of LT_4_ on thyroid hormones (THs) in serum and hippocampus. The time course of THs concentration after a single systemic administration of LT_4_ (15 μg/kg, I.P.) in **(A)** serum and **(B)** hippocampus. **(C)** Left: rats were intraperitoneally injected with LT_4_ immediately after fear conditioning and carried out dependent 1-day, 7-day, 14-day memory tests; Middle: the learning curve during fear conditioning training; Right: contextual fear memory tests 1 day, 7 days and 14 days after training. LT_4_ group, rats injected with LT_4_; Control group, rats injected with saline. Numbers in bars represent animal numbers. I.P., intraperitoneal injection; H, hour; D, day; LT_4_, levothyroxine; FT_4_, free tetraiodothyronine; FT_3_, free triiodothyronine. *P < 0.05, ^&^*P* < 0.01, ^#^*P* < 0.001.

To examine the effects of systemic injection of LT_4_ on the level of free THs in serum and hippocampus, rats were received a single LT_4_ administration (15 μg/kg, I.P.). The data with one-way ANOVA analysis showed that the serum FT_4_ and FT_3_ was significantly increased from 2 h to 3 days and returned to baseline 7 days after administration (Figure 1A, FT_4_: *F*_(4,15)_ = 163.26, *P* < 0.001; *post hoc*: 2 h, *P* < 0.001; 1 day, *P* < 0.001; 3 days, *P* < 0.001; 7 days, *P* > 0.99, respectively, compared with control group. FT_3_: *F*_(4,15)_ = 39.81, *P* < 0.001; *post hoc*: 2 h, *P* < 0.001; 1 day, *P* < 0.001; 3 days, *P* < 0.05; 7 days, *P* > 0.99, respectively, compared with control group; *n* = 4 in each time point). Meanwhile, the hippocampal FT_4_ and FT_3_ was also increased from 2 h to 1 day after LT_4_ administration in one-way ANOVA analysis (Figure 1B, FT_4_: *F*_(4,15)_ = 7.47, *P* = 0.005; *post hoc*: 2 h, *P* < 0.01; 1 day, *P* = 0.04; 3 days, *P* = 0.36; 7 days, *P* > 0.99, respectively, compared with control group. FT_3_: *F*_(4,15)_ = 6.21, *P* = 0.009; *post hoc*: 2 h, *P* > 0.99; 1 day, *P* = 0.019; 3 days, *P* > 0.99; 7 days, *P* > 0.99, respectively, compared with control group).

To examine the effects of systemic injection of LT_4_ on fear memory formation, rats were injected with LT_4_ (15 μg/kg, I.P.) immediately after fear conditioning (Figure 1C, left). There was no significant difference in the fear memory acquisition between control group and LT_4_-injection group represented by the learning curve (Figure 1C, middle: repeated measures ANOVA, *F*_(1,14)_ = 0.18, *P* = 0.67), which indicated that the acquisition of fear conditioning between two groups was similar to each other. However, during the following dependent fear memory tests, the rats injected with LT_4_ showed the decreased freezing level compared to control group (Figure 1C, right: repeated measures ANOVA, *F*_(1,14)_ = 14.09, *P* = 0.002; *post hoc*: 1 day, *P* = 0.009; 7 days, *P* < 0.001; 14 days, *P* < 0.001, respectively, compared with control group), which indicated that LT_4_ administration (I.P.) immediately after fear conditioning prevented the maintenance of long-term memory.

Thus, systemic administration (I.P.) of LT_4_ increased the FT_4_ and FT_3_ levels in serum and hippocampus, and impaired long-term fear memory maintenance.

### The Effects of Hippocampal Administration of LT_4_ on FT_4_ and FT_3_ in Hippocampus, Prefrontal Cortex and Serum and on Locomotor Activity and Thigmotaxis

**Figure 2:**
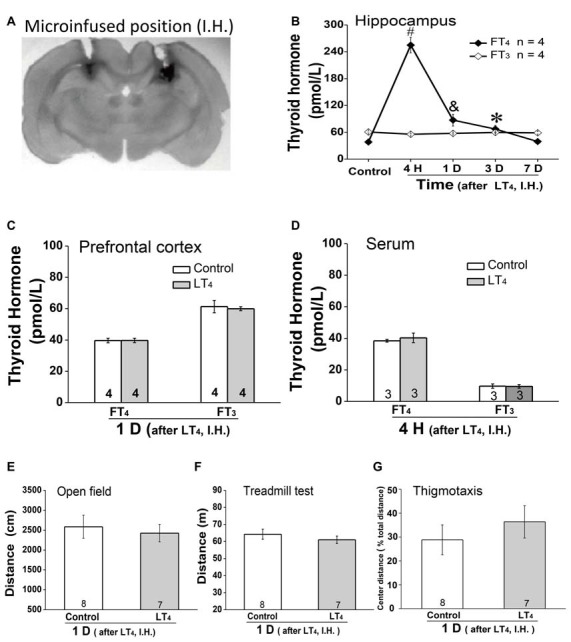
Effects of intrahippocampal injection of LT_4_ on free THs in hippocampus, prefrontal cortex (PFC) and serum, locomotor activity and thigmotaxis. **(A)** The position of the trypan blue-stained area was the histological demonstration of cannula tracts and the typical location for delivery of LT_4_ to hippocampus. **(B)** The time course of hippocampal THs concentration after hippocampal injection of LT_4_ (0.4 μg/μl, 1 μl/side). **(C)** The THs concentration in PFC 1 day after hippocampal injection of LT_4_. **(D)** The THs concentration in serum 4 h after hippocampal injection of LT_4_. **(E)** The open field test 1 day after hippocampal injection of LT_4_. **(F)** The treadmill test 1 day after hippocampal injection of LT_4_. **(G)** The thigmotaxis 1 day after hippocampal injection of LT_4_. Numbers in bars represent animal numbers. I.H., intrahippocampus; PFC, prefrontal cortex; H, hour; D, day; LT_4_, levothyroxine; FT_4_, free tetraiodothyronine; FT_3_, free triiodothyronine. *P < 0.05, ^&^*P* < 0.01, ^#^*P* < 0.001.

As systemic injection of LT_4_ induced dramatical changes of THs in hippocampus, we hypothesized that the hippocampal THs have critical roles on some processes. To address this question, rats were directly injected with LT_4_ into DH. All of our experimental rats for drugs administration in hippocampus were histologically verified the identical loci of injection needle tips in DH and the patency of all tracts for drugs infusion, which was represented by a trypan blue-stained coronal brain from a rat having ever been injected LT_4_ into the DH (Figure 2A; Bradley et al., 1992). The results showed that only FT_4_ but not FT_3_ was increased in hippocampus after LT_4_ injection (Figure 2B, FT_4_: one-way ANOVA, *F*_(4,15)_ = 113.79, *P* < 0.001; *post hoc*: 4 h, *P* < 0.001; 1 day, *P* = 0.007; 3 days, *P* < 0.05; 7 days, *P* > 0.99, respectively, compared with control group. FT_3_: one-way ANOVA, *F*_(4,15)_ = 1.41, *P* = 0.28), in which the contribution of the local conversion of FT_4_ to FT_3_ was similar to the previous study (van Doorn et al., 1983). The increased concentration of FT_4_ in hippocampus post LT_4_ injection lasted at least 3 days and recovered to normal level 7 days later. Meanwhile, a single intrahippocampal LT_4_ injection did not change the level of THs in PFC (Figure 2C, FT_4_: control group vs. LT_4_ group, *t* = 0.03, *P* = 0.97; FT_3_: control group vs. LT_4_ group, *t* = 0.33, *P* = 0.75), which was similar to the previous document (van Doorn et al., 1985). After the above LT_4_ injection, FT_4_ and FT_3_ in serum also did not increase (Figure 2D, FT_4_: control group vs. LT_4_ group, *t* = 0.86, *P* = 0.42; FT_3_: control group vs. LT_4_ group, *t* = 0.09, *P* = 0.92).

In addition, intra-hippocampal injection of LT_4_ did not affect the locomotor activity represented by the total distance in open field test (Figure 2E, control group vs. LT_4_ group, *t* = 0.42, *P* = 0.67), the fatigue resistance ability represented by the total distance in treadmill test (Figure 2F, control group vs. LT_4_ group, *t* = 0.86, *P* = 0.40), and the thigmotaxis represented by the percentage of the TDM in the central zone of the test apparatus (Figure 2G, control group vs. LT_4_ group, *t* = −0.806, *P* = 0.44) which was similar to the previous document (Wilcoxon et al., 2007).

Taken together, LT_4_ infusion in hippocampus selectively increased hippocampal FT_4_ but not FT_3_ concentration in rats. The high level of hippocampal FT_4_ did not change the locomotor activity, the fatigue resistance ability and thigmotaxis.

### The Effects of Hippocampal Administration of LT_4_ on the Stages of Contextual Fear Memory Process

**Figure 3:**
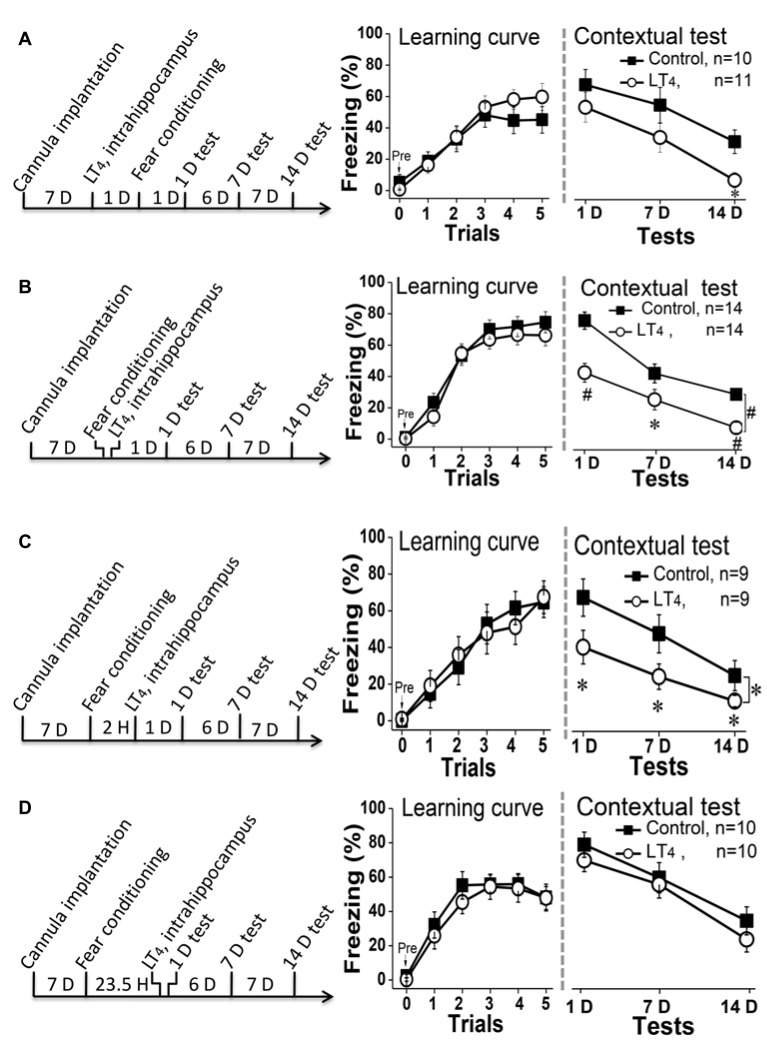
Effects of hippocampal injection of LT_4_ on contextual fear memory. **(A)** Left: hippocampal injection of LT_4_ 1 day before fear conditioning training; Middle: the learning curve during fear conditioning training; Right: contextual fear memory tests 1 day, 7 days and 14 days after training. **(B)** Left: hippocampal injection of LT_4_ immediately after fear conditioning; Middle: the learning curve during fear conditioning training; Right: contextual fear memory tests. **(C)** Left: hippocampal injection of LT_4_ 2 h after fear conditioning; Middle: the learning curve during fear conditioning training; Right: contextual fear memory tests. **(D)** Left: hippocampal injection of LT_4_ 30 min before contextual fear memory test; Middle: the learning curve during fear conditioning training; Right: contextual fear memory tests. LT_4_, levothyroxine; H, hour; D, day. *P < 0.05, ^#^*P* < 0.001.

To test the effects of high level of hippocampal THs on contextual fear memory acquisition, rats were directly injected with LT_4_ into DH through the cannula 1 day before fear conditioning and then carried out dependent memory tests on the day 1, day 7 and day 14 post fear conditioning (Figure 3A, left). The results indicated that the high level of hippocampal THs did not impair the fear memory acquisition represented by the learning curve during the conditioning (Figure 3A, middle. Repeated measures ANOVA, *F*_(1,19)_ = 0.47, *P* = 0.49). However, a single LT_4_ injection impaired the contextual fear memory maintenance because the rats injected with LT_4_ in bilateral hippocampus showed the decreased tendency of freezing level during dependent tests (Figure 3A, right. Repeated measures ANOVA, *F*_(1,19)_ = 3.40, *P* = 0.08; *post hoc*: 1 day, *P* = 0.34; 7 days, *P* = 0.19; 14 days, *P* = 0.012, respectively, compared with control group). This data suggested that the abnormal high level of hippocampal THs might impair contextual fear memory consolidation.

To confirm this hypothesis, rats were injected with LT_4_ into hippocampus immediately (Figure 3B, left) or 2 h (Figure 3C, left) after conditioning. The data showed that rats injected with LT_4_ in hippocampus indeed revealed being decreased freezing time during the dependent contextual fear memory tests using repeated measures ANOVA (Figure 3B, right; *F*_(1,26)_ = 17.46, *P* < 0.001; *post hoc*: 1 day, *P* < 0.001; 7 days, *P* = 0.026; 14 days, *P* < 0.001, respectively, compared with control group; Figure 3C, right; *F*_(1,16)_ = 3.49, *P* = 0.049; *post hoc*: 1 day, *P* = 0.014; 7 days, *P* = 0.047; 14 days, *P* = 0.016, respectively, compared with control group), although all of these rats had similar fear learning ability represented by the learning curve using repeated measures ANOVA (Figure 3B, middle; *F*_(1,26)_ = 0.87, *P* = 0.35; Figure 3C, middle; *F*_(1,16)_ < 0.001, *P* = 0.98).

Finally, to further exclude that this result was due to that the abnormal high level of hippocampal THs impaired fear memory retrieval, we extended the intrahippocampal injection time of LT_4_ to 23.5 h after fear conditioning, so that the changes of 1-D memory test should be used to reflect the roles of LT_4_ injection on memory retrieval (Figure 3D, left). The data suggested that rats injected with LT_4_ in hippocampus showed similar level of learning curve in repeated measures ANOVA (Figure 3D, middle; *F*_(1,18)_ = 0.38, *P* = 0.54) and dependent memory tests whatever the time post conditioning (Figure 3D, right; *F*_(1, 18)_ = 2.26, *P* = 0.15; *post hoc*: 1 day, *P* = 0.94; 7 days, *P* = 0.56; 14 days, *P* = 0.07, respectively, compared with control group). Thus, the high level of hippocampal THs did not impair the contextual fear memory retrieval.

Taken together, the hippocampal LT_4_ injection selectively impaired contextual fear memory consolidation, but not acquisition and retrieval.

## Discussion

In the present study, we found that administration of LT_4_ in hippocampus immediately or 2 h after training impaired the long-term memory of fear conditioning, but pretest or posttraining administration did not affect the acquisition or retrieval. These findings demonstrate that the consolidation of fear memory rather than acquisition or retrieval was affected by hippocampal LT_4_ administration, which suggests that THs receptors or THs in hippocampus may be the modulator of fear memory. Montero-Pedrazuela et al. (2011) have found that fear acquisition was not impaired, fear memory was enhanced, memory extinction was delayed, and spontaneous recovery of fear memory was exacerbated in adult-thyroidectomized Wistar rats. Contextual fear memory was enhanced in a hypothyroid mouse model, and supplementation with T_3_ or T_4_ 2 weeks before training blocked the enhancement of fear memory (Buras et al., 2014). Consistent with these results, we showed that a single systemic or hippocampal administration of LT_4_ impaired fear memory through memory consolidation impairment, which demonstrated for the first time that increased THs in hippocampus can impair fear memory. However, rats were administered T_3_ into the dorsal hippocampal before and after training both exhibited significantly increased long-term memory in contextual fear conditioning (Sui et al., 2006), which may result from the difference of drug administration and fear conditioning protocol.

In clinic, many patients with abnormal THs related diseases such as hypothyroidism and hyperthyroidism, are accompanied by memory impairments (Jabłkowska et al., 2008; Taşkin et al., 2011; de-Miranda et al., 2016; Shrestha et al., 2016). However, the improvements of impaired cognitive functions in patients are often ignored, as many patients still show cognitive disorders even after the THs returns to normal (Sui et al., 2006; Samuels, 2008; Jaracz et al., 2012), which indicates that the abnormal THs level could trigger long-term effects on cognitive functions. These may be due to that THs can regulate synaptic plasticity or molecules identified as mediators for memory processing (Raymaekers and Darras, 2017), including adult neurogenesis (Kapoor et al., 2012, 2015), brain-derived neurotropic factor (BDNF; Yu et al., 2015), glutamic acid decarboxylase 65 (GAD65), neuron-specific K^+^/Cl^−^ co-transporter (KCC2; Sawano et al., 2013). THs can regulate the balance between synaptic LTP and long-term depression (LTD), which play an important role in memory process (Johansen et al., 2011). Most studies have addressed the effects of THs on synaptic plasticity following long-lasting treatments. Impaired LTP has been reported in LT_4_ treated rats (Pavlides et al., 1991; Taşkin et al., 2011) or hypothyroidism rats (Alzoubi et al., 2009; Artis et al., 2012). Hypothyroidism facilitates LTD (Alzoubi et al., 2007) while adult-onset hyperthyroidism causes a durable LTD in perforant pathway-dentate gyrus synapses in rats (Tan et al., 2016). One study has reported that acute intra-hippocampal infusion of LT_4_ can promote LTD over LTP via the integrin αvβ3 receptor (Bitiktas et al., 2017). The effects of THs on the synaptic plasticity and neuronal function maybe the mechanisms underlying the effect of hippocampal administration of LT_4_ on fear memory process.

Limbic regions including hippocampus and amygdala are involved in the circuitry of contextual fear conditioning (Phillips and LeDoux, 1992; Fang et al., 2017; Hegde et al., 2017) and sensitive to hormonal alterations. THs receptors are rich in both hippocampus and amygdala (Puymirat et al., 1991; Desouza et al., 2005; Singh et al., 2016). Stressors induced significant increase in the activity of type 2 deiodinase, which catalyzes T_4_ to the active T_3_ (Baumgartner et al., 1998). Montero-Pedrazuela et al. (2011) reported that abnormal glucocorticoid signaling in the amygdala is an important pathophysiological mechanism underlying fear memory disorder in hypothyroidism. Hippocampus is only involved in fear conditioning situations involving complex, polymodal events, and plays a sensory relay role in fear conditioning. The critical roles of hippocampus in the processes of learning and memory have been generally accepted in human and animal studies (Winocur et al., 2010; Shin and Jadhav, 2016). THs in hippocampus may regulate memory processes through the regulated genes encoding many proteins involved in intracellular signaling pathways. This hypothesis has been partially proved by some previous studies. Hypothyroidism in early postnatal rats triggers a decrease in spatial memory by decreasing hippocampal synaptic plasticity (Salazar et al., 2017). Rats treated with T_3_ showed impaired spatial memory and synaptic LTP induction in hippocampus (Pavlides et al., 1991). LT_4_ daily intraperitoneally injected for 3 months restored impaired spatial memory in aged mice (Fu et al., 2014). However, the effects of hippocampal LT_4_ administration on the different stages of contextual fear memory are unknown.

In this study, single systemic administration of LT_4_ in rats rapidly increased the level of FT_4_ and FT_3_ in serum, which maintained at least for 3 days and returned to normal state post 7 days, which was similar to our previous study (Yu et al., 2015). Interestingly, along with increased level of serum LT_4_, the concentration of FT_4_ and FT_3_ in hippocampus were also increased, which suggested that THs may produce effects on hippocampus related functions such as hippocampus dependent memory. Furthermore, a single systemic LT_4_ administration after fear conditioning dramatically impaired fear memory even when the FT_3_ and FT_4_ returned to normal state (7 and 14 days after LT_4_ administration), which indicates the important role of hippocampal THs in fear memory process.

The roles of hippocampal THs on the stages of contextual fear memory were investigated by hippocampal injection of LT_4_. Our results showed after hippocampal injection of LT_4_, the level of FT_4_ but not FT_3_ increased in the hippocampus (van Doorn et al., 1985). Furthermore, the level of THs in PFC and serum did not changed, which was beneficial to focus studying on the influence of FT_4_ in hippocampus on fear memory. Compared with control group, rats received hippocampal LT_4_ injection 1 day before training showed contact learning and long-term memory detected on 1-day and 7-days, which demonstrated that the increased level of hippocampal THs did not affect contextual fear memory acquisition. However, the impaired 14-days long-term memory indicated the maintenance of long-term memory was influenced in hippocampal LT_4_ injection group. To address the role of hippocampal LT_4_ injection on the fear memory consolidation process, rats were injected with LT_4_ into hippocampus immediately or 2 h after conditioning, and these rats showed sustained long-term memory impairments from 1 day to 14 days. It was consistent with the results in systemic administration of LT_4_ immediately after fear conditioning. In contrast, hippocampal LT_4_ injection 30 min before 1-day test had no effect on the long-term memory, which demonstrated that the increased level of hippocampal THs did not affect fear memory retrieval. Thus, hippocampal administration of THs selectively prevented the consolidation of contextual fear memory. Furthermore, hippocampal LT_4_ injection did not change the locomotor activity and thigmotaxis in open field, and THs in serum or PFC, which might exclude the effect of other factors on memory. Further investigations studying the roles of THs on memory processes in different brain regions will be useful for understanding the mechanisms underlying the cognitive impairments induced by abnormal THs level.

## Conclusion

In summary, our findings suggest that hippocampal administration of LT_4_ selectively impairs the consolidation of contextual fear memory in rats. THs modulation of aversive memories in hippocampus could be involved in etiology of emotional symptoms in THs dysfunction patients. The detailed understanding the role of THs in memory processes will provide mechanisms underlying contribution of THs to memory disorders.

## Author Contributions

DY, HZ and LZ contributed equally to this work. This study was designed by DY, RM and LX; experiments in this study were performed by DY, HZ, LZ, YJ, XW, LJ, QZ and YY; data were analyzed by DY, HZ and LZ; this article was written by DY, HZ and LZ; the design and experiments of this study were supervised by LX and RM; DY, HZ and RM revised the manuscript.

## Conflict of Interest Statement

The authors declare that the research was conducted in the absence of any commercial or financial relationships that could be construed as a potential conflict of interest.

## References

[B1] Alzoubi K. H. Aleisa A. M. Alkadhi K. A. (2007). Adult-onset hypothyroidism facilitates and enhances LTD: reversal by chronic nicotine treatment. Neurobiol. Dis. 26, 264–272. 10.1016/j.nbd.2007.01.002 17331737

[B2] Alzoubi K. H. Gerges N. Z. Aleisa A. M. Alkadhi K. A. (2009). Levothyroxin restores hypothyroidism-induced impairment of hippocampus-dependent learning and memory: behavioral, electrophysiological, and molecular studies. Hippocampus 19, 66–78. 10.1002/hipo.20476 18680156

[B3] Artis A. S. Bitiktas S. Taskin E. Dolu N. Liman N. Suer C. (2012). Experimental hypothyroidism delays field excitatory post-synaptic potentials and disrupts hippocampal long-term potentiation in the dentate gyrus of hippocampal formation and Y-maze performance in adult rats. J. Neuroendocrinol. 24, 422–433. 10.1111/j.1365-2826.2011.02253.x 22070634

[B4] Baumgartner A. Hiedra L. Pinna G. Eravci M. Prengel H. Meinhold H. (1998). Rat brain type II 5′-iodothyronine deiodinase activity is extremely sensitive to stress. J. Neurochem. 71, 817–826. 10.1046/j.1471-4159.1998.71020817.x 9681474

[B5] Beydoun M. A. Beydoun H. A. Rostant O. S. Dore G. A. Fanelli-Kuczmarski M. T. Evans M. K.. (2015). Thyroid hormones are associated with longitudinal cognitive change in an urban adult population. Neurobiol. Aging 36, 3056–3066. 10.1016/j.neurobiolaging.2015.08.002 26329688 PMC4609609

[B6] Bitiktas S. Tan B. Kavraal S. Yousef M. Bayar Y. Dursun N.. (2017). The effects of intra-hippocampal L-thyroxine infusion on long-term potentiation and long-term depression: a possible role for the v3 integrin receptor. J. Neurosci. Res. 95, 1621–1632. 10.1002/jnr.23985 27862211

[B7] Bradley D. J. Towle H. C. Young W. S. III (1992). Spatial and temporal expression of α- and β-thyroid hormone receptor mRNAs, including the β 2-subtype, in the developing mammalian nervous system. J. Neurosci. 12, 2288–2302. 1607941 10.1523/JNEUROSCI.12-06-02288.1992 PMC6575910

[B8] Buras A. Battle L. Landers E. Nguyen T. Vasudevan N. (2014). Thyroid hormones regulate anxiety in the male mouse. Horm. Behav. 65, 88–96. 10.1016/j.yhbeh.2013.11.008 24333846

[B9] Cheng S. Y. Leonard J. L. Davis P. J. (2010). Molecular aspects of thyroid hormone actions. Endocr. Rev. 31, 139–170. 10.1210/er.2009-0007 20051527 PMC2852208

[B10] Chiu K. Lau W. M. Lau H. T. So K. F. Chang R. C. (2007). Micro-dissection of rat brain for RNA or protein extraction from specific brain region. J. Vis. Exp. 7:269. 10.3791/269 18989440 PMC2565859

[B11] Davis P. J. Goglia F. Leonard J. L. (2016). Nongenomic actions of thyroid hormone. Nat. Rev. Endocrinol. 12, 111–121. 10.1038/nrendo.2015.205 26668118

[B12] de-Miranda A. S. Kuriyama S. N. da-Silva C. S. do-Nascimento M. S. Parente T. E. Paumgartten F. J. (2016). Thyroid hormone disruption and cognitive impairment in rats exposed to PBDE during postnatal development. Reprod. Toxicol. 63, 114–124. 10.1016/j.reprotox.2016.05.017 27233481

[B13] Desouza L. A. Ladiwala U. Daniel S. M. Agashe S. Vaidya R. A. Vaidya V. A. (2005). Thyroid hormone regulates hippocampal neurogenesis in the adult rat brain. Mol. Cell. Neurosci. 29, 414–426. 10.1016/j.mcn.2005.03.010 15950154

[B14] Fang T. Kasbi K. Rothe S. Aziz W. Giese K. P. (2017). Age-dependent changes in autophosphorylation of α calcium/calmodulin dependent kinase II in hippocampus and amygdala after contextual fear conditioning. Brain Res. Bull. 134, 18–23. 10.1016/j.brainresbull.2017.06.012 28648815 PMC5599619

[B15] Fu A. L. Zhou R. M. Xu X. R. (2014). The synthetic thyroid hormone, levothyroxine, protects cholinergic neurons in the hippocampus of naturally aged mice. Neural Regen. Res. 9, 864–871. 10.4103/1673-5374.131602 25206902 PMC4146262

[B16] Ge J. F. Xu Y. Y. Li N. Zhang Y. Qiu G. L. Chu C. H.. (2015). Resveratrol improved the spatial learning and memory in subclinical hypothyroidism rat induced by hemi-thyroid electrocauterization. Endocr. J. 62, 927–938. 10.1507/endocrj.EJ15-0253 26228795

[B17] Hegde P. O’Mara S. Laxmi T. R. (2017). Extinction of contextual fear with timed exposure to enriched environment: a differential effect. Ann. Neurosci. 24, 90–104. 10.1159/000475898 28588364 PMC5448453

[B18] Hoermann R. Midgley J. E. Larisch R. Dietrich J. W. (2015). Homeostatic control of the thyroid-pituitary axis: perspectives for diagnosis and treatment. Front. Endocrinol. (Lausanne) 6:177. 10.3389/fendo.2015.00177 26635726 PMC4653296

[B19] Hoermann R. Midgley J. E. Larisch R. Dietrich J. W. (2016). Relational stability of thyroid hormones in euthyroid subjects and patients with autoimmune thyroid disease. Eur. Thyroid. J. 5, 171–179. 10.1159/000447967 27843807 PMC5091265

[B20] Jabłkowska K. Karbownik-Lewińska M. Nowakowska K. Junik R. Lewiński A. Borkowska A. (2008). Working memory and executive functions in hyperthyroid patients with Graves’, disease. Psychiatr. Pol. 42, 249–259. 19697530

[B21] Jaracz J. Kucharska A. Rajewska-Rager A. Lacka K. (2012). Cognitive functions and mood during chronic thyrotropin-suppressive therapy with L-thyroxine in patients with differentiated thyroid carcinoma. J. Endocrinol. Invest. 35, 760–765. 10.3275/8013 21986400

[B22] Johansen J. P. Cain C. K. Ostroff L. E. LeDoux J. E. (2011). Molecular mechanisms of fear learning and memory. Cell 147:948 10.1016/j.cell.2011.10.034 PMC3215943 22036561

[B23] Kapoor R. Desouza L. A. Nanavaty I. N. Kernie S. G. Vaidya V. A. (2012). Thyroid hormone accelerates the differentiation of adult hippocampal progenitors. J. Neuroendocrinol. 24, 1259–1271. 10.1111/j.1365-2826.2012.02329.x 22497336

[B24] Kapoor R. Fanibunda S. E. Desouza L. A. Guha S. K. Vaidya V. A. (2015). Perspectives on thyroid hormone action in adult neurogenesis. J. Neurochem. 133, 599–616. 10.1111/jnc.13093 25772646

[B25] Mahmoud G. S. Amer A. S. (2014). Co-application of corticosterone and growth hormone upregulates NR2B protein and increases the NR2B:NR2A ratio and synaptic transmission in the hippocampus. Sultan Qaboos Univ. Med. J. 14, e486–e494. 25364551 PMC4205060

[B26] Miller K. J. Parsons T. D. Whybrow P. C. van Herle K. Rasgon N. van Herle A.. (2006). Memory improvement with treatment of hypothyroidism. Int. J. Neurosci. 116, 895–906. 10.1080/00207450600550154 16861154

[B27] Mokhtari T. Akbari M. Malek F. Kashani I. R. Rastegar T. Noorbakhsh F.. (2017). Improvement of memory and learning by intracerebroventricular microinjection of T3 in rat model of ischemic brain stroke mediated by upregulation of BDNF and GDNF in CA1 hippocampal region. Daru 25:4. 10.1186/s40199-017-0169-x 28202057 PMC5312580

[B28] Montero-Pedrazuela A. Fernández-Lamo I. Alieva M. Pereda-Pérez I. Venero C. Guadano-Ferraz A. (2011). Adult-onset hypothyroidism enhances fear memory and upregulates mineralocorticoid and glucocorticoid receptors in the amygdala. PLoS One 6:e26582. 10.1371/journal.pone.0026582 22039511 PMC3200331

[B29] Pavlides C. Westlind-Danielsson A. I. Nyborg H. McEwen B. S. (1991). Neonatal hyperthyroidism disrupts hippocampal LTP and spatial learning. Exp. Brain Res. 85, 559–564. 10.1007/bf00231740 1915710

[B31] Phillips R. G. LeDoux J. E. (1992). Differential contribution of amygdala and hippocampus to cued and contextual fear conditioning. Behav. Neurosci. 106, 274–285. 10.1037/0735-7044.106.2.274 1590953

[B32] Puymirat J. Miehe M. Marchand R. Sarlieve L. Dussault J. H. (1991). Immunocytochemical localization of thyroid hormone receptors in the adult rat brain. Thyroid 1, 173–184. 10.1089/thy.1991.1.173 1822365

[B33] Raymaekers S. R. Darras V. M. (2017). Thyroid hormones and learning-associated neuroplasticity. Gen. Comp. Endocrinol. 247, 26–33. 10.1016/j.ygcen.2017.04.001 28390960

[B35] Salazar P. Cisternas P. Codocedo J. F. Inestrosa N. C. (2017). Induction of hypothyroidism during early postnatal stages triggers a decrease in cognitive performance by decreasing hippocampal synaptic plasticity. Biochim. Biophys. Acta 1863, 870–883. 10.1016/j.bbadis.2017.01.002 28088629

[B36] Samuels M. H. (2008). Cognitive function in untreated hypothyroidism and hyperthyroidism. Curr. Opin. Endocrinol. Diabetes Obes. 15, 429–433. 10.1097/MED.0b013e32830eb84c 18769215

[B37] Sawano E. Takahashi M. Negishi T. Tashiro T. (2013). Thyroid hormone-dependent development of the GABAergic pre- and post-synaptic components in the rat hippocampus. Int. J. Dev. Neurosci. 31, 751–761. 10.1016/j.ijdevneu.2013.09.007 24076339

[B38] Shin J. D. Jadhav S. P. (2016). Multiple modes of hippocampal-prefrontal interactions in memory-guided behavior. Curr. Opin. Neurobiol. 40, 161–169. 10.1016/j.conb.2016.07.015 27543753 PMC5056827

[B39] Shrestha S. Bloom M. S. Yucel R. Seegal R. F. Rej R. McCaffrey R. J.. (2016). Thyroid function and neuropsychological status in older adults. Physiol. Behav. 164, 34–39. 10.1016/j.physbeh.2016.05.037 27221367

[B40] Singh S. Rana P. Kumar P. Shankar L. R. Khushu S. (2016). Hippocampal neurometabolite changes in hypothyroidism: an *in vivo* ^1^H magnetic resonance spectroscopy study before and after thyroxine treatment. J. Neuroendocrinol. 28. 10.1111/jne.12399 27203419

[B41] Sui L. Wang F. Liu F. Wang J. Li B. M. (2006). Dorsal hippocampal administration of triiodothyronine enhances long-term memory for trace cued and delay contextual fear conditioning in rats. J. Neuroendocrinol. 18, 811–819. 10.1111/j.1365-2826.2006.01480.x 17026530

[B43] Tan B. Bitiktas S. Kavraal S. Dursun N. Dönmez Altuntas H. D. Suer C. (2016). Low-frequency stimulation induces a durable long-term depression in young adult hyperthyroid rats: the role of p38 mitogen-activated protein kinase and protein phosphatase 1. Neuroreport 27, 640–646. 10.1097/WNR.0000000000000589 27128724

[B44] Taşkin E. Artis A. S. Bitiktas S. Dolu N. Liman N. Süer C. (2011). Experimentally induced hyperthyroidism disrupts hippocampal long-term potentiation in adult rats. Neuroendocrinology 94, 218–227. 10.1159/000328513 21778690

[B45] van Doorn J. Roelfsema F. van der Heide D. (1985). Concentrations of thyroxine and 3,5,3′-triiodothyronine at 34 different sites in euthyroid rats as determined by an isotopic equilibrium technique. Endocrinology 117, 1201–1208. 10.1210/endo-117-3-1201 4017962

[B46] van Doorn J. van der Heide D. Roelfsema F. (1983). Sources and quantity of 3,5,3′-triiodothyronine in several tissues of the rat. J. Clin. Invest. 72, 1778–1792. 10.1172/jci111138 6630526 PMC370467

[B47] Wheeler S. M. McLelland V. C. Sheard E. McAndrews M. P. Rovet J. F. (2015). Hippocampal functioning and verbal associative memory in adolescents with congenital hypothyroidism. Front. Endocrinol. (Lausanne) 6:163. 10.3389/fendo.2015.00163 26539162 PMC4610202

[B48] Wilcoxon J. S. Nadolski G. J. Samarut J. Chassande O. Redei E. E. (2007). Behavioral inhibition and impaired spatial learning and memory in hypothyroid mice lacking thyroid hormone receptor α. Behav. Brain Res. 177, 109–116. 10.1016/j.bbr.2006.10.030 17129617 PMC1819397

[B49] Williams G. R. (2008). Neurodevelopmental and neurophysiological actions of thyroid hormone. J. Neuroendocrinol. 20, 784–794. 10.1111/j.1365-2826.2008.01733.x 18601701

[B50] Willoughby K. A. McAndrews M. P. Rovet J. F. (2014). Accuracy of episodic autobiographical memory in children with early thyroid hormone deficiency using a staged event. Dev. Cogn. Neurosci. 9, 1–11. 10.1016/j.dcn.2013.12.005 24462783 PMC6989760

[B51] Winocur G. Moscovitch M. Bontempi B. (2010). Memory formation and long-term retention in humans and animals: convergence towards a transformation account of hippocampal-neocortical interactions. Neuropsychologia 48, 2339–2356. 10.1016/j.neuropsychologia.2010.04.016 20430044

[B52] Yu D. Zhou H. Yang Y. Jiang Y. Wang T. Lv L.. (2015). The bidirectional effects of hypothyroidism and hyperthyroidism on anxiety- and depression-like behaviors in rats. Horm. Behav. 69, 106–115. 10.1016/j.yhbeh.2015.01.003 25623236

[B53] Zhang W. Song L. Yin X. Zhang J. Liu C. Wang J.. (2014). Grey matter abnormalities in untreated hyperthyroidism: a voxel-based morphometry study using the DARTEL approach. Eur. J. Radiol. 83, e43–e48. 10.1016/j.ejrad.2013.09.019 24161779

[B54] Zhou H. Zhou Q. Xu L. (2016). Unilateral hippocampal inactivation or lesion selectively impairs remote contextual fear memory. Psychopharmacology 233, 3639–3646. 10.1007/s00213-016-4394-7 27485536

